# *KMT2C* mutation is a diagnostic molecular marker for primary thyroid osteosarcoma: A case report and literature review

**DOI:** 10.3389/fmed.2022.1030888

**Published:** 2022-11-08

**Authors:** Xinpei Wang, Qianqian Wang, Peng Su, Chunyan Chen, Bo Han, Zhiyan Liu

**Affiliations:** ^1^Department of Pathology, Shanghai Sixth People’s Hospital Affiliated to Shanghai Jiao Tong University School of Medicine, Shanghai, China; ^2^The Key Laboratory of Experimental Teratology, Ministry of Education, Department of Pathology, School of Basic Medical Sciences, Shandong University, Jinan, China; ^3^Department of Pathology, Qilu Hospital, Shandong University, Jinan, China

**Keywords:** thyroid, osteosarcoma, SATB2, *KMT2C*, mutation

## Abstract

Primary thyroid osteosarcoma is an extremely rare tumor which is associated with a poor prognosis. In this study, we describe an additional case. A 4.5 × 3.8 cm irregular heterogeneous nodule was examined in the left thyroid gland of a 72-year-old woman. Cytological smears and histopathological specimens showed typical features of osteosarcoma with a neoplastic lesion rich in spindle cells with occasional multinucleated cells and lace-like osteoid matrix. Negative immunoreaction with epithelial markers and positive immunoreaction with SATB2 and low Ki-67 labeling index suggested the diagnosis of osteosarcoma. Multiple *KMT2C* gene mutations determined by next-generation sequencing further confirmed the diagnosis.

## Introduction

**FIGURE 1:**
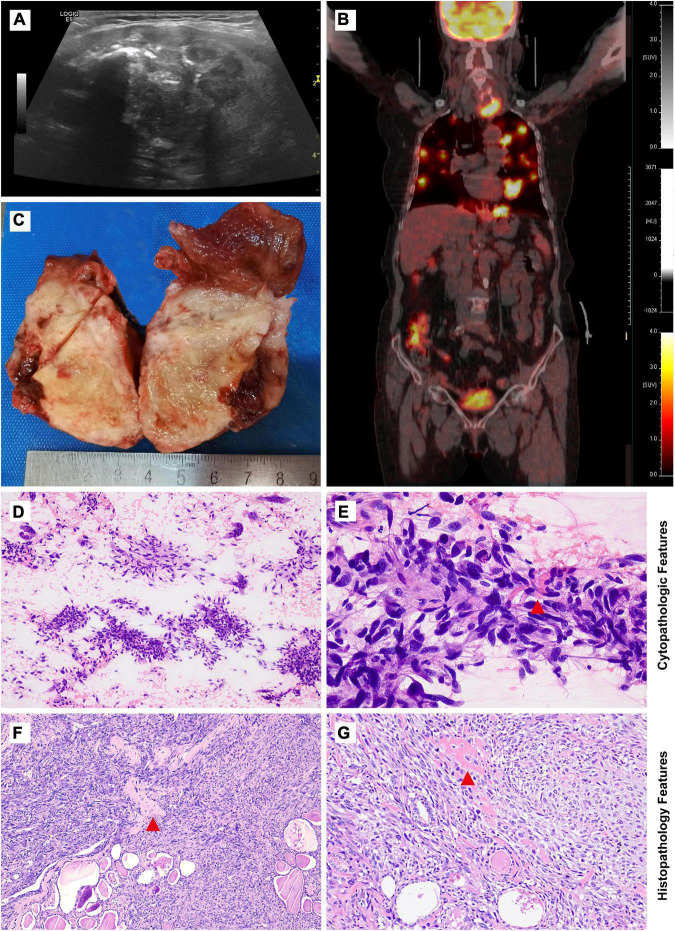
Imaging studies, macroscopic, cytological, and histological features of thyroid osteosarcoma. **(A)** Ultrasonic examination revealed an irregular heterogeneous nodule in the left lobe of the thyroid. **(B)** PET-CT scan showed increased metabolism of FDG in the nodle. **(C)** Grey-yellow nodule infiltrating normal thyroid tissue could be seen grossly. **(D,E)** Cytological features from low magnification in **(D)** and high magnification in **(E)**. **(F,G)** Morphological features from low magnification in **(F)** and high magnification in **(G)**. Red tangle: Pink lace-like osteoid matrix in cell smear and tissue section.

A 72-year-old woman presented with complaints of a painless rapidly growing mass in the neck. No family history of thyroid cancer was documented. Ultrasonic examination revealed a 4.5 × 3.8 cm irregular heterogeneous nodule in the left lobe with multiple patchy calcifications and strong echoes (TI-RADS: 4) without lymph node involvement (Figure 1A). PET-CT scan showed increased metabolism of FDG in the left thyroid lobe (maximum cross section: 4.5 × 4.2 cm; SUVmax: 17.2) with highly heterogeneous density and calcification foci. Multiple masses with high uptake of FDG were observed in the lung (maximum lung window: 4.0 × 2.2 cm; SUVmax: 21.0) but not in the other organs including bones, joints, and soft tissue (Figure 1B).

## Case presentation

### Cytopathologic and histopathological features

UG-FNA was performed. Cell smear specimen was investigated to be rich in atypical spindle tumor cells with a few multi-nucleated cells and mitosis. No necrosis or inflammation was observed in the background. A pink lace-like osteoid matrix suggested the diagnosis of osteosarcoma (Figures 1D,E, Supplementary Figure 2). A total thyroidectomy was performed with a gray or yellow section cutting plane (Figure 1C). Morphologically, the tumor is composed of infiltrative diffuse spindle cells, sparse multi-nucleated giant cells, and an osteoid matrix, which indicated differentiation toward osteosarcoma (Figures 1F,G). No tumor thrombi and necrosis were identified.

### Immunohistochemical features

**FIGURE 2:**
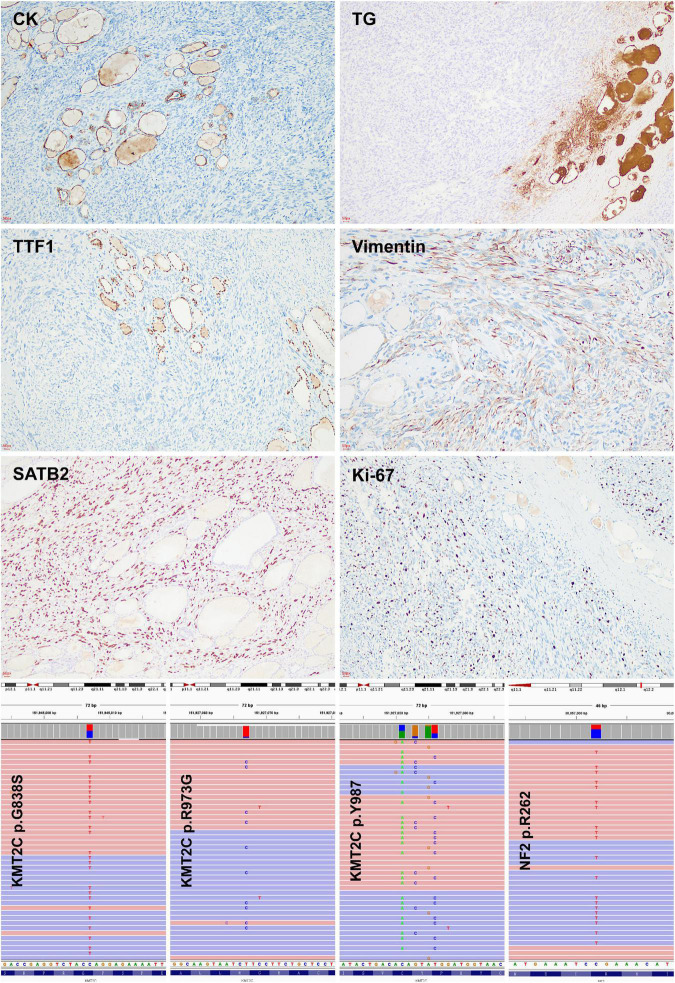
Immunohistochemical and molecular features of thyroid osteosarcoma. Negative immunoreaction with cytokeratin, thyroglobulin, and TTF-1 indicated the non-thyroid follicular origination of the tumour. Positive immunoreaction with SATB-2 and low ki67 labeling index suggested the diagnosis of osteosarcoma. KMT2C mutations were detected by targeted next generation sequencing.

The tumor cells were positive for SATB2, vimentin, and negative for cytokeratin, calcitonin, chromogranin A, synaptophysin, TTF-1, TG, S100, ALK, CD1α, CD34, and CD99 (Figure 2, Supplementary Figure 1). The Ki-67 labeling index ranges between 10 and 15%, which is unusual in anaplastic thyroid carcinoma (ATC) (nearly always higher than 30%).

### Molecular feature

A targeted next-generation sequencing using a 128-gene panel disclosed pathogenic mutations of *KMT2C* (G838S, Y987*, R973G) and *NF2* R262* (Figure 2), but not *BRAF, RAS, RET, TP53*, and *TERT* promoter alterations (Supplementary Table 1).

### Treatment and patient outcome

A total thyroidectomy followed by four courses of first-line chemotherapy regimen of cisplatin and doxorubicin (AP regimen). The patient died of pulmonary metastasis 11 months after surgery.

## Discussion

Osteosarcoma occurs most commonly in the long bones of teenagers and young adults. The extraosseous form of osteosarcoma accounts for less than 5% of all osteosarcomas, especially in the malignant non-epithelial neoplasms of the thyroid and has been reported in only several cases (1–10). Clinically, primary osteosarcoma of the thyroid presents as an aggressive, rapidly growing thyroid mass with a poor prognosis (4). The diagnosis of this rare entity has always been a challenge, for which an overall evaluation must be performed. Here, we report primary osteosarcoma of the thyroid with clinicopathological, cytopathologic, immunohistochemical, and molecular characteristics. Although morphological features have been discussed in the previous studies (1–4), there have been no reports on molecular characteristics of thyroid malignancy with osteoid differentiation. This report is helpful for a better understanding of primary thyroid osteosarcoma and provides the basis for clinical precision therapies.

For the literature review, PubMed and CNKI were searched using the terms: Thyroid Osteosarcoma. The search covered publications in the past century. Only studies describing primary tumors were considered and 11 full-length articles were identified. Only gender/age and final diagnosis were described in some cases, whereas thorough information regarding these cases is limited. Availability of detailed information regarding the reviewed cases is presented in Table 1. The cases consisted of 9 females and 2 males (1–10). The age distribution ranges from 40 to 82 years, which is an older group considering the conventional osteosarcoma. This tumor is characterized by fast growth with pressure-related symptoms of dyspnea and is highly aggressive with invading the surrounding tissue (3). Radical thyroidectomy was performed, combined with postoperative local radiotherapy and/or systemic chemotherapy, for the highly malignant and local recurrence rate. And it is prone to hematogenous metastasis to the lungs, but rarely to cervical lymph nodes. Most patients die of superior vena cava syndrome caused by pulmonary metastasis and extensive invasion of large vessels in the neck. It has been diagnosed in only two cases with fine needle aspiration biopsy and immunohistochemical analysis, but there is no information with molecular characteristics (4, 8). For patients, timely diagnosis and immediate postoperative radiotherapy and chemotherapy can prolong survival time.

**TABLE 1:** Previous studies of primary thyroid osteosarcoma.

References	Gender	Age	Ultra-sound	CT/PET-CT	HE	FNA	IHC	Prognosis
Syrjänen (8)	Female	62	-	-	-	-	-	-
Nitzsche et al. (7)	Female	78	+	+	-	-	-	Lung metastatic disease developed within 12 months.
Strohschneider et al. (6)	Male	82	-	-	-	-	-	-
Hertel et al. (5)	Female	69	-	+	+	-	-	-
Tong et al. (4)	Female	60	+	+	+	+	+	The patient developed a large pulmonary embolus and superior vena cava syndrome and died 5 weeks after the initial diagnosis.
Makis et al. (2)	Female	40	-	+	-	-	-	The patient had developed new lung metastases after treatment with tracheostomy, radiation therapy, and two cycles of chemotherapy.
Zembala-Nożyńska and Lange (3)	Female	76	-	-	+	-	+	The patient remains under oncological and endocrinological care until the last follow-up. Survival time unknown.
Ren et al. (1)	Female	59	+	+	+	-	-	The patient under chemotherapy and radiotherapy and had survived more than 25 months to the last follow-up.
Zhang et al. (15)	Male	62	-	-	+	-	-	The patient had developed recurrence after 6 months treated with radical thyroidectomy.
Wang et al. (16)	Female	68	+	+	+	-	+	The patient treated for thyroidectomy. Survival time unknown.
Lu et al. (17)	Female	40	+	+	+	-	+	The patient treated with thyroidectomy. Survival time unknown.

+: Relevant records are included in the literature. -: No relevant records included in the literature.

Study images showed no lesions in the bones and joints, which supported the thyroid to be the primary site of the tumor in the present case. SATB2 is an emerging immunohistochemical marker of osteoblastic differentiation (Supplementary Figure 2), which is used frequently in the diagnosis of osteosarcoma. The differential diagnosis includes ATC, medullary thyroid carcinoma (MTC), and mesenchymal tumors, in which osteoid and/or cartilage tissue differentiation is occasionally found. ATC could be ruled out morphologically and immunohistochemically of the relatively low Ki-67 labeling index and negative cytokeratin. Negative immunoreaction with neuroendocrine markers, calcitonin, and TTF-1 was helpful to rule out MTC. No expression of S100, CD1α, ALK, CD34, and CD99 was helpful in excluding follicular dendritic cell sarcoma, inflammatory myofibroblastic tumor, angiosarcoma, and synovial sarcoma.

*KMT2C* encodes histone methyltransferase, which regulates gene transcription by modifying chromatin structure. It is essential for embryonic development and cell proliferation. *KMT2C* mutations were observed in 87% of osteosarcoma and may be related to the carcinogenesis of osteosarcoma, among which R973G somatic mutation was the most common variant (11). Mutated *KMT2C* can modify its cooperation with estrogen receptors and then affect bone remodeling and matrix production (11–13). In the current case, four variants of *KMT2C* mutations were detected, including R973G, and no alteration of *BRAF, RAS*, and *RET* genes was found, which indicated that it is not a follicular cell-derived thyroid carcinoma but osteosarcoma (14). *KMT2C* mutation should be related to the tumorigenesis of thyroid osteosarcoma.

In summary, we demonstrated *KMT2C* mutation in thyroid osteosarcoma for the first time. Given the wide morphologic spectrum of osteosarcoma and the propensity to mimic other spindle cell neoplasms, osteosarcoma should be considered in the differential diagnosis of poorly differentiated and anaplastic thyroid malignancies with osteoid differentiation. The osteoid matrix and SATB2 are useful characteristics to confirm the diagnosis. The finding of *KMT2C* mutation in thyroid osteosarcoma suggested the possibility of same driver mutation in extraosseous and intraosseous osteosarcomas, which may have important targeted therapy implications.

## Data availability statement

The original contributions presented in this study are included in the article/Supplementary material, further inquiries can be directed to the corresponding author.

## Ethics statement

The study was conducted in accordance with the Declaration of Helsinki and approved by the Ethical Committee of the School of Basic Medical Science of Shandong University (protocol code ECSBMSSDU2019-1-024). Informed consent was obtained from all subjects involved in the study. Written informed consent has been obtained from the patient to publish this study.

## Author contributions

ZL: conceptualization, methodology, software, formal analysis, investigation, resources, project administration, and funding acquisition. XW: writing—original draft preparation and visualization. ZL and BH: validation and supervision. ZL and PS: data curation. XW and QW: writing—review and editing. All authors read and agreed to the published version of the manuscript.
