# Supplementary material for: KMT2C mutation is a diagnostic molecular marker for primary thyroid osteosarcoma: A case report and literature review
Source: Front Med (Lausanne). 2022 Nov 8;9:1030888. doi: 10.3389/fmed.2022.1030888 (PMC9679279; doi:10.3389/fmed.2022.1030888)
Supplement: Supplementary file 1 [file Data_Sheet_1.DOCX]

Supplementary Material

# Materials and Methods

## Cell smear and tissue sections

Ultrasound-guided FNA was applied as a preoperative diagnosis of thyroid nodules. Direct cell smears of fine-needle aspiration were fixed with 95% ethyl alcohol, and surgical samples were fixed in neutral formalin. Cell smears and paraffin-embedded tissue sections were stained with haematoxylin-eosin. These slides were evaluated blindly by two independent pathologists (Dr. Peng Su and Prof. Zhiyan Liu). Osteosarcomas tissue was used as a positive control for the morphological feature of paraffin-embedded tissue.

## Immunohistochemical staining

Dissected thyroid and tumor tissues were formalin-fixed paraffin-embedded. Paraffin section specimens were immunostained. The sections were incubated using the monoclonal antibodies CK, TG, TTF1, Vimentin, Ki-67, S100, ALK, ß-catenin, CD1A, CD34, CD68 and CD99 obtained from GmbH Zsbio (Beijing, China), Ventana GmbH (Tucson, AZ, USA), Osteosarcomas was used as a positive control for SATB2 (Figure S1).

## Bioinformatics analysis

Sequencing libraries were generated using the thyroid cancer-related NGS panel (RigenBio) according to the manufacturer’s instructions. Genomic DNA and total RNA were isolated from thyroid FFPE tissues using AllPrep DNA/RNA FFPE Kit (QIAGEN). Total RNA was firstly reverse-transcribed into cDNA. An aliquot of DNA or cDNA was subjected to multiplex amplification of target regions, followed by the addition of unique dual index and Illumina sequencing adaptor through PCR amplification. After bead purification, the indexed libraries were quantitated on a Qubit fluorometer (Thermo Fisher), and then sequenced for 150bp paired-end reads on NovaSeq 6000 platform (Illumina). Raw sequencing reads were trimmed to remove adapters and low-quality bases with Cutadapt (v1.18). The processed reads were aligned to the human reference genome hg19 using BWA (v0.7.17). VarScan2 (v2.4.4) was used for SNV/InDel calling, and the variants were annotated using Ensembl Variant Effect Predictor (VEP). Gene fusions were detected based on the fusion transcript sequences using a customized script.

# Supplementary Figures


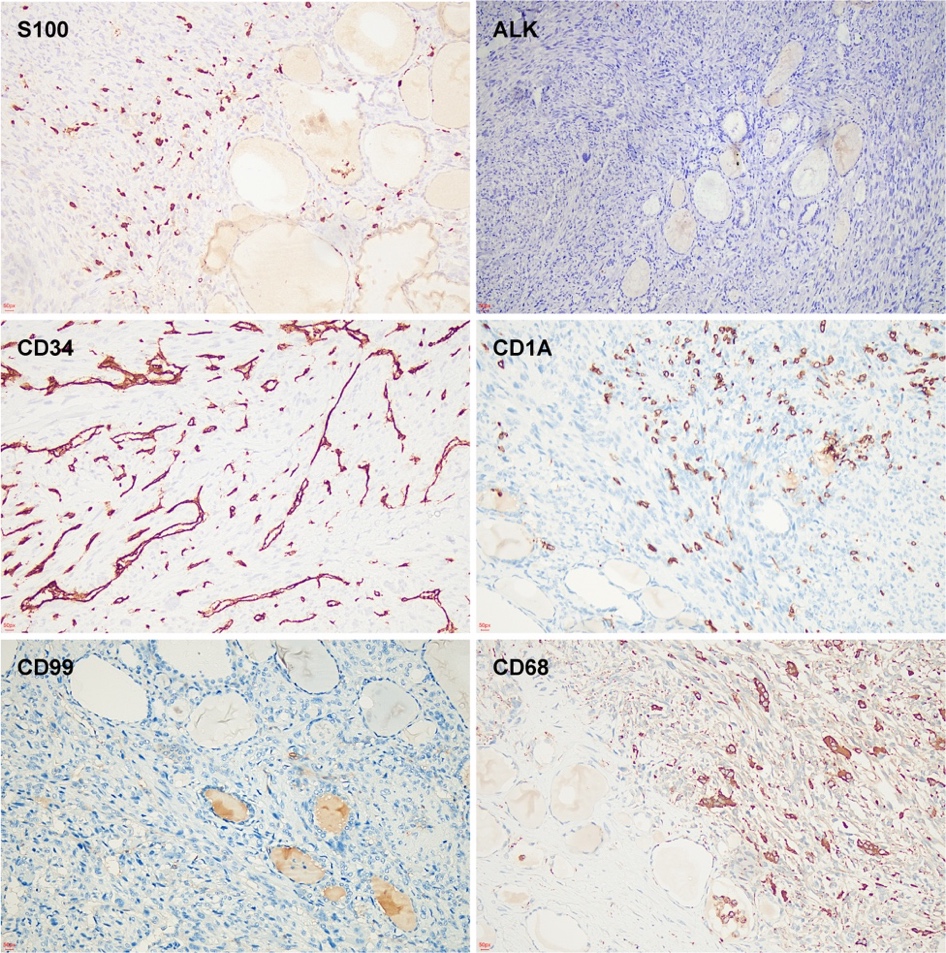


**Supplementary Figure 1.** IHC for differential diagnosis. CD68 was found to be positive in osteoclasts. S100 and CD1α were expressed in individual dendritic cells but not tumor cells. Positive immunoreaction with ß-catenin in the cytoplasm of the carcinoma cells. Negative immunoreaction with ALK, CD34 and CD99 in tumor cells.


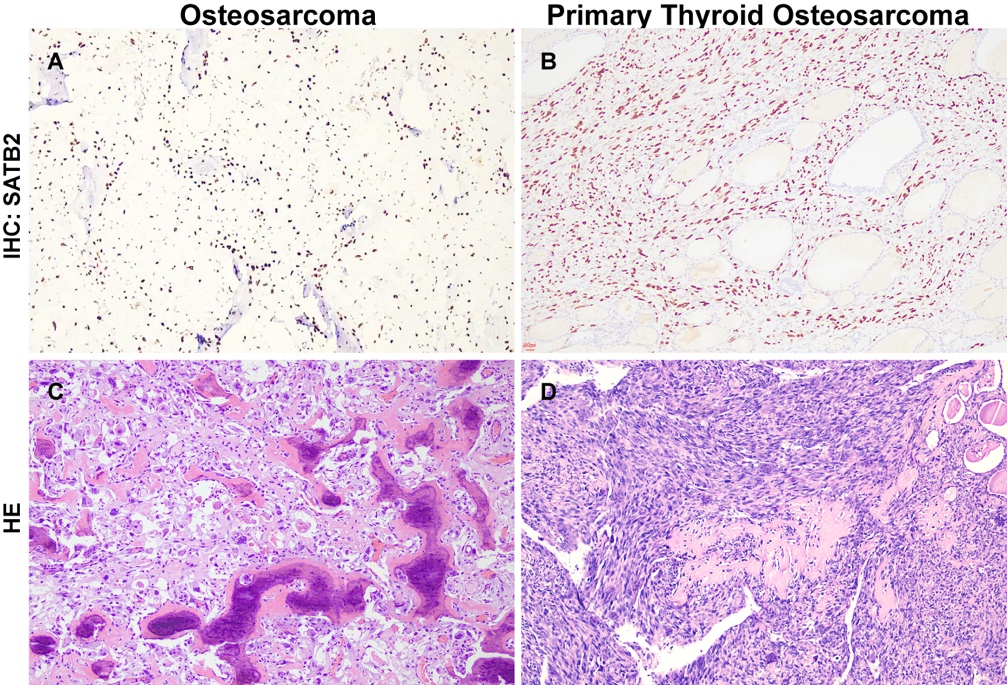


**Supplementary Figure 2.** Osteosarcoma was used as a positive control to primary thyroid osteosarcoma. (A, B) Immunoreaction with SATB2 of osteosarcoma (A) and primary thyroid osteosarcoma (B). (C, D) Histopathology feature showed that spindle tumor cells with moderate to severe atypia with diffuse distribution of osteoid matrix and multinucleated giant cells.

# Supplementary Table

**Supplementary Table.** The molecular features of this primary thyroid osteosarcoma.

| **Gene** | **HGVSc** | **HGVSp** | **Mutation Type** | **Mutation Frequency** | **COSMIC Number/**  **Rs Number** |
| --- | --- | --- | --- | --- | --- |
| KMT2C | NM_170606.2:c.2512G>A | NP_733751.2:p.G838S | missense variant | 46.16% | COSM4591269 |
| KMT2C | NM_170606.2:c.2961C>G | NP_733751.2:p.Y987* | stop gained | 15.08% | COSM216053/rs58528565 |
| KMT2C | NM_170606.2:c.2959T>C | NP_733751.2:p.Y987H | missense variant | 10.89% | COSM253767 |
| KMT2C | NM_170606.2:c.2917A>G | NP_733751.2:p.R973G | missense variant | 10.14% | COSM4161993 |
| NF2 | NM_000268.3:c.784C>T | NP_000259.1:p.R262* | stop gained | 36.13% | COSM22000/rs74315496 |
| NF1 | NM_001042492.2:c.2951G>A | NP_001035957.1:p.G984E | missense variant | 9.61% | COSM6264623 |
| TSC2 | NM_000548.4:c.806G>A | NP_000539.2:p.G269D | missense variant | 1.31% | COSM6368925 |
| ATM | NM_000051.3:c.7318A>G | NP_000042.3:p.K2440E | missense variant | 0.77% | COSM6191555/rs1565529306 |
| ATM | NM_000051.3:c.8122G>A | NP_000042.3:p.D2708N | missense variant | 0.51% | COSM48246/rs587782719 |
| FGFR4 | NM_213647.2:c.769G>A | NP_998812.1:p.A257T | missense variant | 0.75% | COSM6405602 |
| SMAD4 | NM_005359.5:c.1082G>A | NP_005350.1:p.R361H | missense variant | 0.74% | COSM14122/rs377767347 |
| SMAD4 | NM_005359.5:c.382G>A | NP_005350.1:p.V128M | missense variant | 0.53% | COSM14210 |
| FARSB | NM_005687.4:c.1333G>A | NP_005678.3:p.A445T | missense variant | 0.58% | COSM6393801 |
| GLIS3 | NM_001042413.1:c.2710G>A | NP_001035878.1:p.G904R | missense variant | 0.51% | rs150310830 |
| KMT2C | NM_170606.2:c.2828C>T | NP_733751.2:p.T943I | missense variant | 45.68% | / |
| KMT2C | NM_170606.2:c.2822A>T | NP_733751.2:p.H941L | missense variant | 45.59% | / |
| KMT2C | NM_170606.2:c.2824A>G | NP_733751.2:p.N942D | missense variant | 45.41% | / |
| ATM | NM_000051.3:c.8768T>C | NP_000042.3:p.V2923A | missense variant | 16.71% | / |
| TSHR | NM_000369.2:c.143C>G | NP_000360.2:p.P48R | missense variant | 49.08% | / |
| LRP1B | NM_018557.2:c.6599T>C | NP_061027.2:p.I2200T | missense variant | 47.91% | / |
| GLIS3 | NM_001042413.1:c.1774A>G | NP_001035878.1:p.T592A | missense variant | 0.52% | / |
